# The Clinical Phenotype of Early Selective Mutism and Later Autism Spectrum Disorder in Girls: A Case Series Analysis

**DOI:** 10.3390/children12020237

**Published:** 2025-02-16

**Authors:** Hagit Nagar Shimoni, Efrat Zilbershot Fink, Yael Leitner

**Affiliations:** Marot Autism Center, Child Development Institute, Dana-Dwek Children’s Hospital, Tel Aviv Sourasky Medical Center, 6 Weizmann St., Tel Aviv 6492403, Israel; efratzf@tlvmc.gov.il (E.Z.F.); leitnery@tlvmc.gov.il (Y.L.)

**Keywords:** selective mutism, autism spectrum disorder, early diagnosis, clinical presentation, girls, masking behaviors, early marker

## Abstract

**Background:** The presentation of autism spectrum disorder (ASD) in girls often differs from that of boys, leading to delayed diagnosis. Selective mutism presenting at a young age can obscure autism symptoms, particularly in girls who exhibit “masking” behaviors. In this study, we examined the early manifestation of selective mutism in four girls who were later diagnosed with ASD. **Method:** The study describes four case studies of girls who underwent an ASD diagnostic process. All were either diagnosed at an early age with selective mutism or had selective mutism suspected as a potential diagnosis during their preschool years. Clinical information was collected through detailed developmental history and clinical evaluations by a multidisciplinary team. **Results:** All girls were diagnosed at a young age with selective mutism by a child psychiatrist and later with ASD by a multidisciplinary team. They all demonstrated normal-range intelligence. This is in agreement with Muris and Steffenburg asserting that in a certain group of children, an early manifestation of selective mutism may be an early marker of ASD. **Conclusions**: Special follow-up should be given to girls diagnosed with selective mutism during their preschool years because this could be an early marker for a later ASD diagnosis.

## 1. Introduction

There has been a growing awareness of the clinical presentation of autism in populations other than boys. A meta-analysis showed a male-to-female ratio of 4.56:1 in studies relying on passive identification of autism based on medical records. However, the ratio narrows to 3.25:1 in studies using active case finding in the general population [1]. The authors note that autism in females is under-recognized, with predictive models suggesting 39% more females should receive a diagnosis than currently identified. Research also indicates that autism in females is often diagnosed later, especially if the assessment occurs beyond preschool age, as mental health diagnoses can obscure or mask autism [2].

Social difficulties, social anxiety, and communication challenges in girls are often not accurately recognized or treated early. Girls tend to exhibit fewer restricted and repetitive behaviors (RRBs) compared to boys, show gender-normative interests, higher social attention, and motivation for friendships, and often engage in “masking” behaviors. These features make diagnostic challenges more pronounced, especially during adolescence when social and functional challenges become more evident [2,3,4,5,6].

Recent studies have suggested a potential overlap between autism spectrum disorder (ASD) and selective mutism (SM) [7,8,9,10,11,12] (see Table S1 in the Supplementary Materials). SM is an anxiety disorder characterized by the inability to speak in specific social situations, despite having normal speaking abilities in other contexts. The prevalence of the disorder is estimated to range between 0.2% and 0.5% of the population [7,13]. Studies indicate that the disorder is more common in girls, with a ratio of nearly 2:1 compared to boys. Other anxiety disorders are also more prevalent in girls compared to boys [7,13].

Concerning the potential overlap of ASD and SM, one study examining the medical records of children diagnosed with SM (n = 97) at an ASD-specialized center reported a mean age of SM onset at 4.5 years and SM diagnosis at 8.8 years. SM was more common in girls (male-to-female ratio of 2.7:1), with 63% of children with SM meeting diagnostic criteria for ASD [7]. Children with both ASD and SM had later symptom onset, delayed SM diagnosis, speech delays, and a higher prevalence of borderline intellectual functioning or intellectual disability. These findings emphasize the diagnostic challenges posed by the intersection of SM and ASD.

Research on the relationship between SM and ASD remains limited, partly due to the artificial boundaries imposed by classification systems. For instance, the DSM-5 [13] specifies that the primary feature of SM—failure to speak—should not occur exclusively in the context of ASD. However, this distinction is challenging in practice, given the dimensional nature of ASD, which blurs the lines between these two conditions [14].

At our diagnostic center, we focus on autism evaluations for children aged 6–18 years old. A total of 314 children were assessed in 2024 for suspected autism spectrum disorder (ASD). Of these, 114 were girls, and 200 were boys. Among the girls, nine were diagnosed with selective mutism at an early age by a child psychiatrist and subsequently diagnosed with ASD by our team. These cases represent 7.89% of all girls referred to our center for communication difficulties in 2024 and 2.87% of all children evaluated for suspected ASD during the same year.

This article presents four case studies of girls aged 7–14 years old, all in mainstream education with normal-range cognition, who were initially diagnosed or suspected of having selective mutism in early childhood and later diagnosed with ASD. These descriptive cases highlight the manifestations of both conditions over time and emphasize the importance of considering SM as an early marker of ASD in girls.

## 2. Materials and Methods

### 2.1. ASD Diagnostic Guidelines in Israel

According to the guidelines of the Israeli Ministry of Health, ASD assessments for children up to the age of 18 require:

1. Evaluation by two professionals: A physician (either a neurologist or psychiatrist) and a licensed psychologist specializing in one of the following fields: clinical, educational, developmental, or rehabilitative psychology.
2. Comprehensive assessment:
  - A thorough physical examination
  - An extensive parental interview
  - A detailed developmental interview
  - A communication assessment by a physician
  - A communication assessment by a psychologist
  - Utilization of at least one standardized autism diagnostic tool, such as ADOS-2 or CARS-2
  - Use of at least one parent-report questionnaire, such as SCQ, SRS-2, or ADI-R
  - Functional assessment using tools such as Vineland or ABAS-2 [15]
  - Cognitive level assessment
3. Consensus between the two professionals (a physician and a psychologist)
4. Involvement of additional professionals as needed based on clinical indications
5. Diagnosis based on DSM-5 criteria

### 2.2. Multidisciplinary Approach at Our Center

Our center follows a multidisciplinary approach, involving various specialists and diagnostic tools to ensure a comprehensive evaluation. The diagnostic team includes:

- Developmental neurologists
- Psychiatrists
- Clinical, educational, and developmental psychologists
- Clinical social workers
- Art therapists with a master’s degree

### 2.3. Roles in the Diagnostic Process

1. Physician’s role:
  - Conducts a parental interview
  - Performs a physical examination
  - Conducts a communication assessment
  - Participates in multidisciplinary team discussions, including neurologists, psychiatrists, clinical, developmental, educational, and rehabilitative psychologists, clinical social workers, and autism-specialized art therapists
  - Total duration: 5 h
2. Psychologist’s role:
  - Conducts two parental meetings
  - Performs a communication assessment
  - Reviews and integrates parent-report questionnaires with clinical data
  - Participates in multidisciplinary team discussions with physicians, psychologists, clinical social workers, and autism-specialized art therapists
  - Total duration: 5 h

Following the medical and psychological assessments, the center provides:

- Summary meetings and diagnostic disclosure: Conducted by the psychologist.
- Group parent counseling: Led by a clinical social worker at the end of the diagnostic process [16].

### 2.4. Participants

- Four girls aged 7–14 years old in mainstream education with normal-range cognition.
- All were initially diagnosed or suspected of having SM during early childhood.
- All the girls were diagnosed with an IQ within the normal range (90–110).
- All the girls had no hearing or vision defects and no history of brain injury.

### 2.5. Data Collection

- Detailed developmental histories provided by caregivers
- Data from medical records, medical examinations, psychologist reports, and past therapeutic and educational reports
- Use of standardized diagnostic tools:
  1. ADOS-2: Autism Diagnostic Observation Schedule
  2. SCQ: Social Communication Questionnaire
  3. ABAS-II: Adaptive Behavior Assessment tool
  4. Neurological evaluation
  5. Psychological evaluation
  6. Detailed report on the child’s academic, social, and emotional functioning at school
  7. DSM 5

### 2.6. Procedures

The girls’ developmental trajectories were examined to explore the co-occurrence and interplay of ASD and SM symptoms. Detailed case studies were compiled to illustrate the presentation and progression of these conditions in girls.

## 3. Results: Case Summaries

### 3.1. Orly (Pseudonym), Age 7

- Age of selective mutism diagnosis: 4.5 years old.
- Age of autism diagnosis: 7 years old.
- Reason for referral: Orly, a 7-year-old girl currently attending a small classroom within a mainstream school, was referred for ASD assessment at the recommendation of a child psychiatrist and a pediatric neurologist. The referral followed ongoing concerns about her social and communication difficulties, selective mutism, repetitive behaviors, and sensory sensitivities, as well as her struggles to engage socially despite her ability to understand learning material.
- Background and early development: Orly was born by cesarean section 38 weeks after an uncomplicated pregnancy, weighing 2900 g, with no complications during delivery or in the neonatal period. Developmental delays were noted early on: she began walking after the age of 2 and required physical therapy. Her first words also emerged after the age of 2. Toilet training was delayed, with the successful transition out of diapers only by age 3.
- Family background: Orly is the third of four children. One sibling is diagnosed with autism. The family has not undergone genetic counseling.
- Early social and emotional characteristics: during preschool, Orly exhibited significant SM, speaking only to specific individuals. She avoided social interactions, did not initiate relationships, and preferred solitary activities. At home, she displayed marked separation anxiety, clinging to her mother when outside and crying without an apparent trigger. These symptoms of anxiety emerged later when she was evaluated at our center.
- Current functioning: Orly is described by her teachers as diligent and responsible, able to understand learning material but struggling to navigate social interactions. She speaks selectively, avoids eye contact, and rarely initiates social engagement, often isolating herself during breaks. She demonstrates repetitive behaviors, such as hand-flapping, strict adherence to routines, and hypersensitivity to noise and fabrics.
- Repetitive and restricted behaviors, sensory regulation challenges: Orly exhibits repetitive hand movements and vocalizations. Her behavior is characterized by rigidity and adherence to routines. She displays sensitivity to noise and specific fabric textures. Her high-intensity areas of interest include drawing and art.
- Cognitive and behavioral assessments:
  1. IQ: Above 90.
  2. ADOS-2: Score 15 (cutoff: 7–8).
  3. SCQ (parent questionnaire): Score 24 (cutoff: 15).
  4. ABAS-II General adaptive functioning: Score 53 (very low).
- Medical examination: clinical observation highlights pronounced selective mutism, difficulties with non-verbal communication, and repetitive, non-reciprocal functional play.
- Psychological assessment: Orly was nonverbal during the evaluation, communicating primarily through eye contact. She did not engage in imaginative play but participated in concrete card games. She repeatedly expressed distress over dirt in the examination room.
- Diagnosis: Based on the assessments, Orly meets the criteria for ASD.

### 3.2. Betty (Pseudonym), Age 12

- Age of selective mutism diagnosis: During preschool (approximately 4–5 years).
- Age of autism diagnosis: 12 years.
- Reason for referral: Betty, a 12-year-old girl currently attending a small classroom within a mainstream school, was referred for ASD assessment at the recommendation of a child psychiatrist. The referral was initiated due to her persistent social and communication difficulties, selective mutism, restricted interests, sensory sensitivities, and recent symptoms of depression and anxiety, including auditory hallucinations. Her low adaptive functioning and challenges in daily tasks further supported the need for this evaluation.
- Background and early development: Betty was born prematurely at 34 weeks, weighing 2000 g, after an otherwise uncomplicated pregnancy. Neonatal development was unremarkable, and she was discharged on time. She began walking at age 1 but has not yet mastered riding a bicycle without training wheels. A child and adolescent psychiatrist diagnosed her with SM at the age of 4.5 years, taking into account her bilingual background—Russian and Hebrew—and her SM is present in both languages. Speech development milestones were slightly delayed, and she was toilet-trained before the age of 3.
- Family background: Betty is the youngest of three siblings, and one of her sisters has been diagnosed with autism. The family has not pursued genetic counseling.
- Early social and emotional characteristics: in preschool, Betty exhibited selective mutism, refraining from speaking to staff and often expressing difficulty understanding Hebrew. She was described as extremely shy with strangers but friendly and open with familiar people, sometimes displaying poor social judgment. Over time, she developed a small circle of friends but continues to struggle with initiating social interactions. Betty prefers interacting with younger children and playing online with a small group of peers.
- Current functioning: Betty has been diagnosed with ADHD, learning disabilities, and selective mutism, and she recently began experiencing symptoms of depression and anxiety. She described hearing the voice of an imaginary friend and is currently treated with sertraline (Lustral). While she enjoys digital animation, computer games, and cats, these interests dominate her activities, often leading to repetitive discussions about these topics. Betty struggles to meet age-appropriate expectations in daily functioning and continues to face significant social difficulties.
- Repetitive and restricted behaviors, sensory regulation challenges: Betty displays repetitive hand movements, echolalia, and a rigid adherence to routines. She has strong interests in specific topics, such as animation and cats, and exhibits sensory sensitivities, particularly to noise and certain fabrics.
- Cognitive and behavioral assessments:
  1. IQ: Above 90.
  2. ADOS-2: Score 13 (cutoff: 7–8).
  3. SCQ (parent questionnaire): Score 12 (cutoff: 15).
  4. ABAS-II General adaptive functioning: 42 (very low).
- Medical examination: Betty presented as physically mature for her age, with partial eye contact and limited facial expressions. While she demonstrated intact emotion recognition and theory of mind, she struggled with understanding complex social situations and exhibited repetitive interest in cats. Cognitive assessments revealed difficulties with abstract language, including idioms and slang, as well as tic-like behaviors involving her nose.
- Psychological assessment: Betty avoided eye contact and showed limited facial expressions but engaged pleasantly on a personal level. She demonstrated word-finding difficulties, grammatical errors, and a preoccupation with cats during the session.
- Diagnosis: Betty meets the criteria for ASD.

### 3.3. Gali (Pseudonym), Age 10

- Age of selective mutism diagnosis: During preschool (approximately 3–4 years).
- Age of autism diagnosis: 10 years.
- Reason for referral: Gali, a 10-year-old girl in a mainstream class with inclusion support, was referred for ASD evaluation at the recommendation of a pediatric neurologist. The referral was prompted by ongoing social challenges, selective mutism, repetitive behaviors, sensory sensitivities, and rigid adherence to routines. Additionally, her limited communication with peers and emotional regulation difficulties highlighted the need for assessment.
- Background and early development: Gali was born at full term (39 weeks), weighing 2900 g, after a healthy pregnancy and delivery. She began walking at 14 months but has not yet learned to ride a bicycle without training wheels. Gali has mild motor clumsiness and received occupational therapy as a result. Her speech development was typical, with her first words appearing by age 1, and no language regression was noted. She was toilet-trained by age 2.
- Family background: Gali is the third of five siblings. Three of her siblings have been diagnosed with autism. The family has not yet pursued genetic counseling.
- Early social and emotional characteristics: Adjusting to preschool was extremely challenging for Gali, marked by episodes of standing by the door crying. She was socially passive, followed instructions as though being “led like a puppet”, and rarely initiated interactions. While she sometimes joined group activities, she preferred interacting with younger children. By preschool age, selective mutism was suspected, as Gali did not speak with her teacher or peers.
- Current functioning: Gali demonstrates repetitive behaviors, such as patterned doll play, and shows significant sensory sensitivities, particularly to noise. Her daily routines are rigid, and she struggles with understanding complex social situations. Gali has limited social interactions and communicates minimally with peers.
- Repetitive and restricted behaviors, sensory regulation challenges: Gali engages in patterned doll play and exhibits rigid adherence to routines. She is notably sensitive to noise and has a soft, quiet voice.
- Cognitive and behavioral assessments:
  1. IQ: Above 90.
  2. ADOS-2: Score 13 (cutoff: 7–8).
  3. SCQ (parent questionnaire): Score 19 (cutoff: 15).
- Medical examination: Gali displayed intermittent eye contact, limited facial expressions, and repetitive movements involving her face, neck, and shoulders. She had difficulty understanding complex social scenarios, primarily identifying basic emotions. Her play was described as rigid and structured.
- Psychological assessment: Gali avoided social interactions, exhibited significant anxiety, and maintained partial eye contact. Her social engagement was minimal, and her playing was idiosyncratic. She refused to separate from her mother, communicated primarily through head gestures, and whispered about having “stage fright”.
- Diagnosis: Gali meets the criteria for ASD.

### 3.4. Dorit (Pseudonym), Age 14

- Age of selective mutism diagnosis: 3 years.
- Age of autism diagnosis: 14 years.
- Reason for referral: Dorit, a 14-year-old girl in a mainstream classroom, was referred for an ASD evaluation at the recommendation of a psychiatrist. The referral was prompted by ongoing challenges, including selective mutism, social withdrawal, sensory sensitivities, rigid behaviors, and limited peer interactions, despite her academic capabilities.
- Background and early development: Dorit was born at 42 weeks, weighing 3200 g, after a healthy pregnancy and delivery. She began walking at 18 months and learned to ride a bicycle without training wheels only at age 13. She exhibited mild motor delays and received occupational therapy for coordination difficulties during preschool. Her speech development was delayed, with her first words appearing at age 2, though no language regression was noted.
- Family background: Dorit is the second of six siblings. Four of her siblings have been diagnosed with autism and anxiety. The family has not yet undergone genetic counseling.
- Early social and emotional characteristics: from the age of 3, Dorit exhibited extreme social anxiety and selective mutism, often hiding under tables in the presence of unfamiliar people, including family members. She was diagnosed with selective mutism and anxiety at the time. In preschool, she was socially withdrawn, did not participate in group activities, and failed to form friendships.
- Current functioning: Dorit is a capable learner and is described as a good student, but she avoids seeking help from teachers, peers, or family members. She struggles with independence in daily life and has limited peer relationships, often preferring younger children. Her activities are focused on a few restricted interests, such as bead-making and drawing. Dorit exhibits notable sensory sensitivities, particularly to noise, and rigid behaviors like adhering to fixed routines.
- Repetitive and restricted behaviors, sensory regulation challenges: Dorit shows a preference for routine and exhibits rigid eating habits. Her interests are narrowly focused on crafts like bead-making and drawing. She also demonstrates significant noise sensitivity.
- Cognitive and behavioral assessments:
  1. IQ: Above 90.
  2. ADOS-2: Score 20 (cutoff: 7–8).
  3. SCQ (parent questionnaire): Score 28 (cutoff: 15).
  4. ABAS-II General adaptive functioning: Score 43 (very low).
- Medical examination: Dorit presented with intermittent eye contact and limited facial expressions. Her behavior demonstrated a strong attachment to routine and significant difficulty understanding complex social situations.
- Psychological assessment: Dorit avoided social interactions and exhibited high levels of anxiety. Her responses were minimal, and she engaged in solitary activities.
- Diagnosis: Dorit meets the criteria for ASD.

### 3.5. Similarities Between the Four Cases

- Selective mutism (SM): All four cases exhibited selective mutism during early childhood, characterized by an inability to speak in specific social settings despite normal speech in other contexts. This shared feature often led to initial diagnoses of anxiety disorders or selective mutism rather than ASD.
- Delayed ASD diagnosis: Despite early developmental concerns, ASD diagnoses were delayed until later childhood or adolescence. These delays often followed prolonged periods of unrecognized social and communication difficulties. This timeline highlights that the diagnosis of selective mutism often occurred earlier than the diagnosis of autism, where selective mutism and autism were identified concurrently during adolescence. The delayed autism diagnoses in most cases underline the challenges in recognizing ASD in girls, particularly when masked by specific clinical profiles of selective mutism.
- Family background: In all cases, at least one sibling of the participant was diagnosed with autism.
- Social challenges: All participants faced significant difficulties forming and maintaining peer relationships. They were described as socially withdrawn, initiating minimal social interactions, and preferring structured or one-on-one engagements.
- Cognitive functioning: All girls had IQ scores above 90, reflecting normal cognitive abilities. However, they demonstrated impairments in adaptive functioning, especially in social domains.
- Restricted and repetitive behaviors (RRBs): Each girl displayed some form of RRBs, such as rigid routines, repetitive movements, or restricted interests (e.g., cats, animation, crafts).
- Sensory sensitivities: Sensory challenges, such as hypersensitivity to noise or certain textures, were common across all cases.
- Overlap of anxiety and autism: All the girls exhibited symptoms of anxiety, with some receiving additional diagnoses of anxiety disorders or depressive episodes, highlighting the comorbidity between anxiety and autism.
- ADOS-2 threshold: All girls met the threshold for autism classification on the ADOS 2 test. This finding contradicts previous data regarding girls with normal-range intelligence and ASD.

### 3.6. Differences Between the Four Cases

- Age at ASD diagnosis: Ehe age of diagnosis varied significantly, ranging from early childhood (age 7) to late adolescence (age 14).
- Language development: Language milestones differed: some participants (e.g., Orly and Dorit) experienced clear speech delays, while others (e.g., Gali) had typical or mildly delayed language development.
- Severity of social impairments: The degree of social impairment ranged from profound withdrawal and minimal peer interaction (e.g., Orly and Dorit) to limited but existent social circles (e.g., Betty).
- Comorbid diagnoses: Additional diagnoses, such as ADHD, learning disabilities, or depression, differed across cases, contributing to variations in clinical complexity.
- Restricted interests: The specific nature of restricted interests varied, ranging from animals (e.g., Betty) to crafts and artistic activities (e.g., Dorit).

### 3.7. Summary of Similarities and Differences

The similarities and differences between the four cases are summarized in Tables S2–S4 (see Supplementary Materials).

## 4. Discussion

These four cases illustrate the diagnostic complexity of ASD in girls, particularly when presenting with SM behaviors at an early age, social challenges, and normal-range intelligence. Our findings underscore several critical issues and considerations for clinical practice.

### 4.1. Diagnostic Challenges in Girls with ASD

- Differential diagnosis: Evaluating older children for autism requires specialized expertise in neurodevelopmental and psychiatric disorders, as well as an understanding of overlapping communication difficulties across different conditions.
- Unique presentation in older children: Autism may manifest differently in older children, necessitating a nuanced approach to diagnosis.
- Gender-specific presentation and masking: Girls with ASD often exhibit a distinct clinical profile compared to boys. They are more likely to camouflage or compensate for their autistic characteristics, which can delay diagnosis [1,2,4,17,18,19]. These masking behaviors make it harder for standardized tools to capture the full scope of their social and communication difficulties.
- Co-occurrence of mental health conditions: A prominent feature of autistic girls is the frequent overlap with mental health conditions such as anxiety, depression, or selective mutism. These conditions can either overshadow the autism diagnosis or, conversely, be overshadowed by it, leading to under-identification or misdiagnosis [2].
- ADOS-2 scoring patterns: While research suggests that many girls with ASD and normal-range intelligence often score below the diagnostic threshold on the ADOS-2 (likely due to masking behaviors), all four cases in this study exceeded the clinical threshold, highlighting the distinct scoring nuances of the ADOS 2 when assessing girls with ASD and early selective mutism presentation [4,17,18,19].

### 4.2. Implications for Practice

- Awareness of early SM presentation and later ASD diagnosis in girls: Given the significant overlap between ASD and SM, particularly in girls, clinicians must consider the possibility of SM as an early marker of a later ASD diagnosis in cognitively able girls. Awareness of this early clinical presentation in girls with social difficulties can help avoid delays in evaluation and intervention and provide a more accurate clinical picture.
- Dual interventions: Effective treatment plans should address both ASD and SM symptomatology. Interventions should include strategies to enhance social and adaptive functioning, reduce anxiety, and support communication needs [7,14]. Current interventions for SM include behavioral therapy, pharmacological treatment, and integrative behavioral therapy [20,21,22]. Autism treatment typically involves a combination of individual and group therapy (using various approaches), parent training, and placement in an appropriate educational setting [23,24,25,26].
- Consideration of gender differences: The diagnostic process should account for gender norms and expectations that may shape the presentation of ASD in girls. Subtler or atypical traits, compared to boys, require clinicians to adapt their frameworks for assessment.
- A comprehensive multidisciplinary assessment integrating the child’s medical history and communication profile and utilizing multiple diagnostic tools should be conducted by multiple healthcare professionals. This diagnostic practice ensures the integration of the clinical presentation from early childhood with the current clinical picture, producing a reliable diagnosis with comprehensive treatment recommendations.

### 4.3. Revisiting the Diagnostic Thresholds

Previous studies suggest that girls with autism and normative intelligence often fail to meet the diagnostic threshold on tools like the ADOS-2, likely due to masking behaviors and subtle presentations of ASD traits [4,9,10,11]. However, the current study challenges this assumption: all four girls in our case series, despite having normative intelligence, exceeded the ADOS-2 diagnostic threshold for ASD. This finding underscores the need for acknowledging the unique presentation in the ADOS-2 of this population.

## 5. Conclusions

Selective mutism (SM) in girls, as we described here, is an important example of the challenges of ASD diagnosis in girls., particularly when presenting at an early age. Despite normative intelligence, all four girls exceeded the ADOS-2 diagnostic threshold, countering existing literature suggesting that girls with ASD and normal intelligence [2,4,17,19] often fail to meet this threshold.

In all cases, SM was the initial diagnosis with ASD identified only later. We reinforce others, e.g., [7,14], in advocating for a more nuanced evaluation and strict follow up of girls presenting with SM in early childhood, as SM may obscure an underlying ASD diagnosis or maybe an early marker of ASD. The four cases presented in this study emphasize the need for a comprehensive and sensitive approach to diagnosis and intervention.

### Limitations

While these findings provide valuable insights into the diagnostic complexities of SM and ASD in girls, the current study has several important limitations that must be acknowledged. These limitations include a very small sample size, an exclusively female participant group, and a SM diagnosis reported by medical records and not by the current team.

We suggest using our case study article as an important addition to Muris and Steffenburg’s research [7,14] and to continue investigating this topic comprehensively by using larger samples of boys and girls, across a wide age range, over time, and with specific questionnaires measuring SM.

## Data Availability

The original contributions presented in this study are included in the article/Supplementary Material. Further inquiries can be directed to the corresponding author.

## Supplementary Materials

The following supporting information can be downloaded at: https://www.mdpi.com/2227-9067/12/2/237/s1, Table S1: Similarities and Differences Between SM and ASD; Table S2: Basic Information and Developmental Background of the Four Girls; Table S3: Development and Function of the Four Girls; Table S4: Social Functioning and Diagnostic Assessment of the Four Girls.
