# Supplementary material for: The Clinical Phenotype of Early Selective Mutism and Later Autism Spectrum Disorder in Girls: A Case Series Analysis
Source: Children (Basel). 2025 Feb 16;12(2):237. doi: 10.3390/children12020237 (PMC11854050; doi:10.3390/children12020237)
Supplement: Supplementary file 1 [file children-12-00237-s001.zip › children-3461220-supplementary.pdf]

Supplementary Materials

Table S1: Similarities and Differences Between SM and ASD

Aspect	Selective Mutism (SM)	Autism Spectrum Disorder (ASD)
**Social Difficulties**	Difficulties in social interactions; individuals with SM tend to avoid speaking in specific social situations.	Difficulties in social interactions; individuals with ASD may feel uncomfortable in social situations.
**Anxiety**	Anxiety is a central component of SM; individuals often experience high anxiety in certain social settings.	Anxiety is common in ASD; individuals may also experience high anxiety, especially in social situations.
**Communication Challenges**	Challenges in communication; SM manifests as an inability to speak in specific environments.	Challenges in communication; difficulties can be broader, affecting comprehension, expression, body language, or intonation.
**Nature of the Condition**	Primarily an anxiety disorder; characterized by difficulty speaking in specific social settings despite normal speech ability elsewhere.	A neurodevelopmental condition affecting multiple domains, including communication, social interaction, and behavior.
**Symptom Range**	The main difficulty is speech inhibition due to anxiety in specific settings.	A broader spectrum of symptoms, including repetitive behaviors, difficulty understanding language and communication, and restricted interests.
**Onset and Early Signs**	Typically appears between ages 3–5 when a child is exposed to new social environments like preschool.	Can be identified as early as the first year of life, often with early communication and behavioral delays.
**Speech Patterns**	Children with SM speak normally in comfortable settings (e.g., at home).	Individuals with ASD may have delayed speech development, atypical language use, or difficulty with conversational reciprocity.
**Underlying Causes**	Primarily linked to social anxiety or environmental-emotional factors.	A combination of genetic, biological, and sometimes environmental factors.

Table S2: Basic Information and Developmental Background of the Four Girls

#	Name	Age	Educational Framework	Medical Background	Birth Weight (g)	Birth Week
1	Orly	7	Small class in regular setting	Normal pregnancy, normal course	2900	38
2	Betty	12	Small class in regular setting	Normal pregnancy, normal course	2000	34
3	Gali	10	Small class in regular setting	Normal pregnancy, normal course	2900	39
4	Dorit	14	Regular class	Normal pregnancy, normal course	3200	42

Table S3: Development and Function of the Four Girls

#	Name	Motor Development	Language Development	Strengths and Challenges
1	Orly	Received physiotherapy	First words after age two	Repetitive movements and sounds, rigid routine
2	Betty	Independent without walking aids	Slightly delayed first words, incomplete mother tongue	Hand flapping, speech anxiety
3	Gali	Received occupational therapy	First words at age one, no intonation	Pattern play with dolls
4	Dorit	Slightly clumsy	Speech delay, first words at age two	Sensitivity to noise, fixed routine

Table S4: Social Functioning and Diagnostic Assessment of the Four Girls

#	Name	Social Characteristics	Family	General Function (ABAS II) Normal range 90-110	SCQ Score Threshold 15	ADOS 2 Score Threshold 7-8
1	Orly	Introverted, difficulty forming connections, selective mutism	One brother with autism	53.0	24	15
2	Betty	Social with adults, shy with strangers	One sister with autism	42.0	12	13
3	Gali	Adaptation difficulties, anxiety, socially passive	Three siblings with autism	Not specified	19	13
4	Dorit	Avoids eye contact, social anxiety	Four siblings with autism and anxiety	43.0	28	20
